# Lack of in-brace x-rays in compliant AIS patients wearing full-time TLSO braces associates with failure

**DOI:** 10.1186/s13018-021-02650-9

**Published:** 2021-08-31

**Authors:** Isabel Alvarez, Kiley Poppino, Lori Karol, Amy L. McIntosh

**Affiliations:** 1 University of Texas-Southwestern, Dallas, TX USA; 2 Texas Scottish Rite Hospital for Children, 2222 Welborn St, Dallas, TX 75219 USA; 3 Children’s Hospital Colorado, Aurora, CO USA

**Keywords:** Adolescent idiopathic scoliosis, In-brace correction

## Abstract

**Background:**

In-brace correction and brace compliance with thoraco-lumbo-sacral orthotic (TLSO) braces are associated with successful treatment of adolescent idiopathic scoliosis (AIS). This paper compares patients who had consistent radiographic documentation of in-brace correction to those who did not.

**Methods:**

All skeletally immature (Risser 0-2) patients were treated for AIS (25-45°) with full-time TLSO braces that had compliance temperature monitors. All patients wore their braces at least 12 h a day. Brace failure was defined as curve progression to a surgical magnitude (≥ 50°). All patients were followed until brace discontinuation.

**Results:**

Ninety patients (F 82, M 8) with an average age of 12.1 (10.1-15.0) years, Risser grade 0 (0-2), BMI percentile 48.5 (0.0-98.8), and daily brace wear of 16.5 (12.1-21.6) h/day were treated for 24.3 (8.0-66.6) months. Patients went through 1.7 (1-4) braces on average. Forty-two out of 90 (46.7%) patients had some amount of brace time with an unknown in-brace correction, which, on average, was 66.1% of their total treatment course (11.5-100). On univariate analysis, patients that did not have a repeat in-brace x-ray with major brace adjustments or new brace fabrication tended to be more skeletally immature (Risser 0 and tri-radiate open, *p* = 0.028), wear more braces throughout their treatment (2.0 vs 1.4, *p* < 0.001), were treated for a longer period of time (27 vs 22 months, *p* = 0.022), and failed bracing more often (47.6% vs 22.9%, *p* = 0.014).

**Conclusions:**

Patients who did not have new in-brace x-rays with major brace adjustments and/or new brace fabrication were 3.1 (95% CI 1.2-7.6) times more likely to fail bracing than patients who were re-checked with new in-brace x-rays.

**Trial registration:**

ClinicalTrials.gov—NCT02412137, initial registration date April 2015

**Level of evidence:**

III

## Background

Adolescent idiopathic scoliosis (AIS), the most common form of idiopathic scoliosis, occurs when there is greater than 10° of curvature of the spine by age 10 years or greater, with no identifiable organic cause [1]. Moderate scoliotic curves are currently treated with a brace—an effective and non-invasive method to decrease curve progression [1–6, 7]. Some of the widely accepted risk factors for curve progression in AIS include female sex, skeletal immaturity determined by Risser/tri-radiate cartilage staging and female menarche status, extreme body mass index (BMI), a greater curve magnitude, and specific curve types such as main thoracic curves [1–6, 8–12].

Brace use and compliance have also become important considerations when assessing the likelihood of brace failure and progression to surgical treatment [4, 5]. It has been shown that a brace dose-response curve exists in AIS patients, in which increasing hours of daily brace wear correlates with increased rates of success. An average of 12.9 h predicted a success rate of 90 to 93% [5]. This study set a compliance threshold of greater than 12 h per day in an effort to increase the sample size of our population studied. Studying a compliant population has become essential to understanding what factors associated with the brace itself are correlated with successful non-operative management. For example, in-brace correction is a value measuring the percent decrease in curve magnitude while the patient wears his or her brace compared to the curve magnitude without the brace. Braces that correct the scoliotic curve by at least 40–50% have been shown to stabilize or improve curves while braces that lead to less than 10% correction have been associated with an increased risk of brace failure [6, 10].

**Fig. 1:**
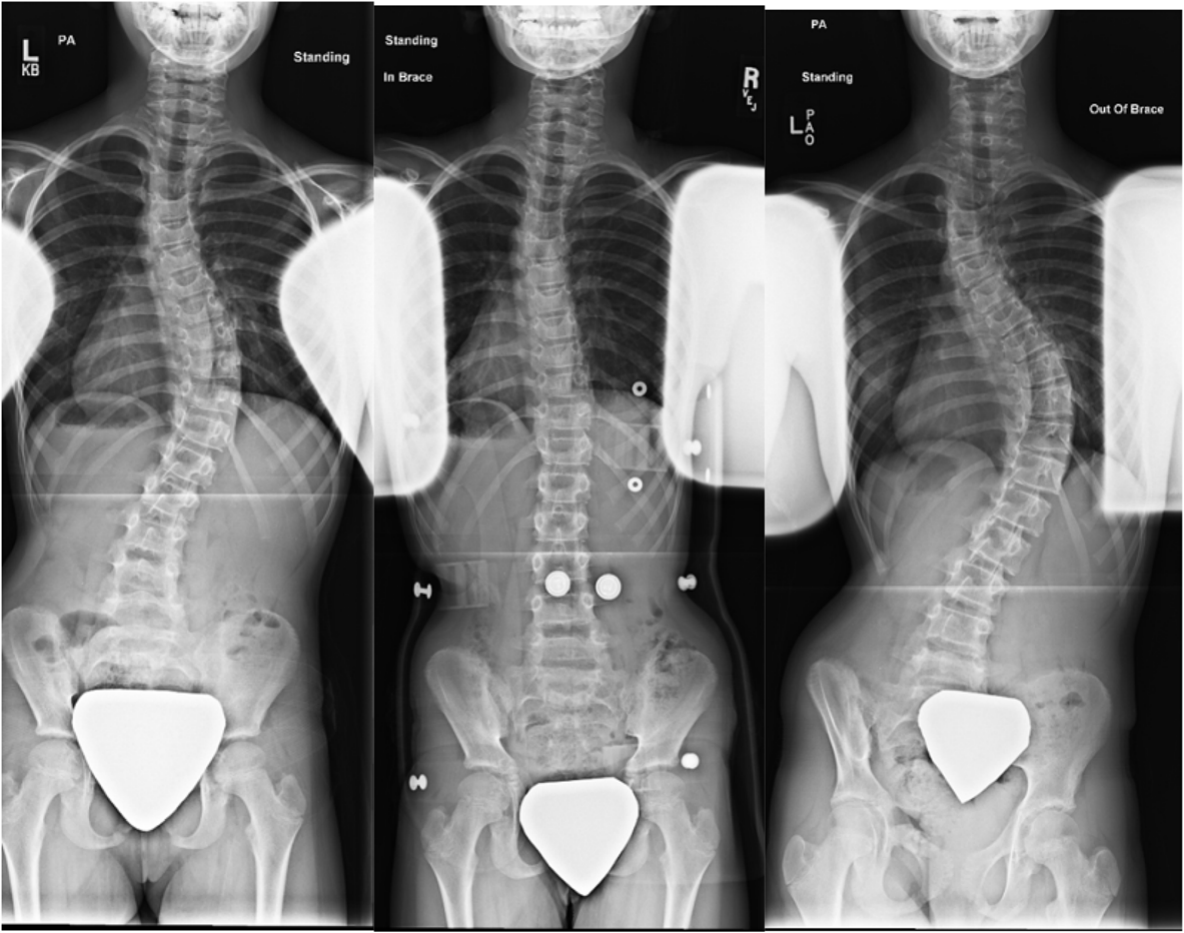
Patient example. This patient is a 10-year-old female who was Risser 0 with open tri-radiate cartilage at brace initiation. Her thoracic curve measured 44° initially (**a**). In her brace, her curve decreased to 9.9°, a 77.6% correction from her original curve (**b**). Thirteen months later, the patient was fitted for a second brace, but new in-brace x-rays were not taken. She was fitted for a third brace 15 months later without confirmation x-rays. This patient was compliant with bracewear; however, for 57.5% of her treatment time, she had an unknown in-brace correction. She ultimately discontinued brace use with a final curve of 63.8°, requiring surgery (**c**)

It has become common practice for bracing studies to only report in-brace correction from the first in-brace radiograph [2–4, 6, 8, 11, 13]. However, it has been empirically identified that many adolescents outgrow their braces and need either large brace adjustments or new brace fabrication entirely (Fig. 1). The in-brace correction of the first brace is then assumed to be the effect throughout the entire treatment course even though adjustments are made throughout. Patients who consistently have in-brace radiographs with each large brace adjustment or new brace fabrication will have more opportunities to optimize radiograph-based risk factors—such as in-brace correction—compared to patients who are inconsistently documented. This paper compares patient characteristics and outcomes between those who had consistent radiographic documentation of in-brace correction throughout their treatment course to those who did not.

## Methods

This was a retrospective review of a prospectively enrolled study of braced AIS patients, consisting of 218 patients enrolled from 2008 to 2013. Patients included in this study were ages 10 to 16 at the start of bracing, were both male and female, and were of any race or ethnicity. All patients were treated at a single institution for AIS and had not worn a brace before, but were prescribed a thoraco-lumbo-sacral orthotic (TLSO) brace once bracing indications were met. The initial curves at diagnosis measured between 25 and 45°. Measurement of Cobb angles on spinal radiographs have several sources of errors, and techniques suggested by Capasso, Maffulli, and Testa were taken to minimize such errors [14]. The initial Risser scores were 0, 1, or 2 and females were premenarchal or less than 1 year post menarche. Patients were compliant with their braces, which were determined by small temperature sensors placed inside the brace.

For this study, patients were excluded where a diagnosis other than AIS was found to explain the scoliotic curves. Other criteria that led to exclusion included initial curves less than 25° or greater than 45°; initial Risser scores of 3, 4, or 5; and females greater than 1 year post menarche. If the patient had surgery before reaching 50°, they were excluded from analysis. Patients without complete temperature sensor data or patients who had a change in treatment during bracewear were excluded. Patients were also excluded if they showed, on average, less than 12 h per day of bracewear determined by the temperature information collected from the brace.

Curve progression was measured by Cobb angle measurements. There were 4 standardized clinic visits with radiographs required by the previous study: brace prescription, brace initiation, brace discontinuation, and final follow-up or preoperative. All clinic visits where patients had documentation of a large brace adjustment or new brace prescription were also recorded. Patients who had at least one clinic visit with a large adjustment or new brace prescription without a new in-brace radiograph were defined as having inconsistent in-brace correction documentation. Patients who had in-brace radiographs with every large brace adjustment or new brace prescription were defined as having consistent in-brace correction documentation.

Successful bracing treatment was defined as both thoracic and either thoracolumbar or lumbar Cobb angles maintaining below 50° at final follow-up. Brace failure was defined as curve progression to greater than or equal to 50° or performance of a spinal fusion surgery.

Statistical analysis was performed. Means and ranges were used to describe continuous variables, and percentages were used for categorical variables. Transformation of variables was used to ensure that normality assumptions were satisfied. Univariate analysis was used to determine what variables significantly correlated with inconsistent in-brace correction documentation. Significance was set at *p* < 0.05.

## Results

**Fig. 2:**
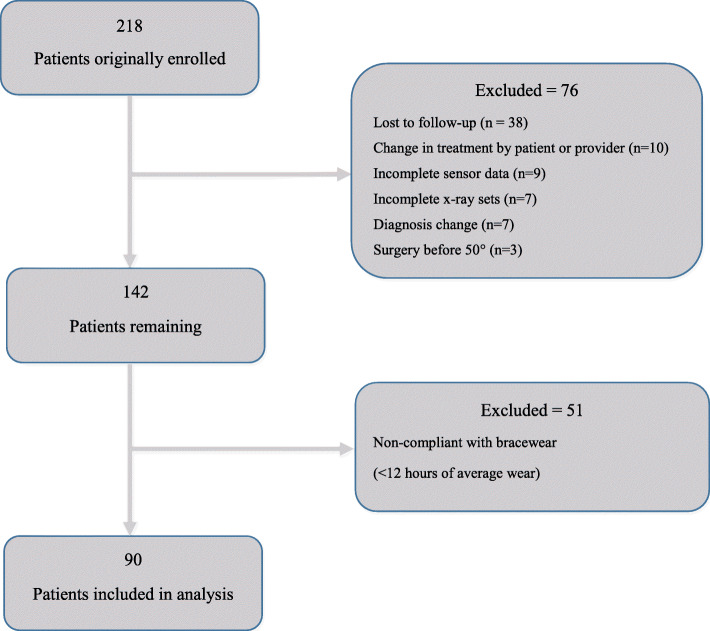
Flow diagram of patient exclusion criteria

There were 218 patients originally enrolled from 2008 to 2013 (Fig. 2). Thirty-eight of these patients were lost to follow-up and another 38 were excluded using the listed exclusion criteria. Of the remaining 142 patients, 51 patients were additionally excluded due to the study’s compliance threshold for an average daily wear of greater than 12 h throughout the entire treatment.

After inclusion and exclusion criteria were applied, 90 patients remained (Table 1). At the brace prescription timepoint, on average, patients were 12.1 years old, Risser grade 0, and BMI percentile 48.5. They wore their braces 16.5 h per day (12.1-21.6) and were treated for 24.3 months (8.0–66.6). Patients required 1.7 braces (1-4) throughout their treatment course, on average.

**Table 1:** Comparison of patients followed consistently with x-rays vs. followed inconsistently

	Consistent (*n* = 48)	Inconsistent (*n* = 42)	*p* value
Age (*years*)	12.4 ± 1.0	11.7 ± 1.1	0.010
BMI percentile	52.2 ± 26.4	44.4 ± 29.2	0.200
Treatment duration (*months)*	22.1 ± 8.8	27.0 ± 10.9	0.022
Brace prescription (*h/day*)	20.3 ± 2.4	20.1 ± 2.2	0.479
Actual bracewear (*h/day*)	16.6 ± 2.8	16.3 ± 2.5	0.677
Major Cobb angle (°)	32.7 ± 5.5	33.8 ± 4.5	0.230
Total number of braces	1.4 ± 0.7	2.0 ± 0.7	< 0.001
Unmonitored correction (*% of treatment duration*)	0.0 ± 0.0	66.1 ± 31.0	< 0.001
Risser 0, tri-radiate open (*%*)	22.9	47.6	0.028
Successful treatment (*%*)	77.1	52.4	0.014

Forty-two of these patients (46.7%) had some amount of treatment time with an unknown in-brace correction, which, on average, was 66.1% of their total treatment course (11.5-100). On univariate analysis, patients with inconsistent in-brace correction documentation tended to be more skeletally immature (Risser 0 and tri-radiate open, *p* = 0.028), wear more braces throughout their treatment (2.0 vs 1.4, *p* < 0.001), were treated for a longer period of time (27 vs 22 months, *p* = 0.022), and failed bracing more often (47.6% vs 22.9%, *p* = 0.014). They were 3.1 (95% CI 1.2-7.6) times more likely to fail bracing than patients who were consistently followed with in-brace x-rays.

## Discussion

During brace treatment for AIS, standing x-rays of the spine should be obtained in standardized manner to provide the best patient care possible, balancing both risks and benefits of the radiation. The standard imaging tool for identifying and monitoring AIS is standing posterioanterior (PA) and lateral scoliosis x-ray films. Positioning the patient in this manner reduces radiation exposure to both the thyroid and breast tissue.

The two main societies clinically dealing with idiopathic scoliosis are the Scoliosis Research Society (SRS), founded in 1966, and the International Society on Scoliosis Orthopedic and Rehabilitation Treatment (SOSORT), started in 2004. In 2014, the SOSORT published a 2012 consensus paper entitled: reducing x-ray exposure in pediatric patients with scoliosis [15]. This group recommended obtaining spinal radiographs every 12 months for patients 13–18 years of age with AIS, Risser stages 0-3. They also recommended that a lateral radiograph be taken during the first assessment of a scoliosis patient and not during every subsequent AP or PA radiograph, unless the patient has a significant sagittal plane deformity that appears to be changing. However, this group was unable to make any specific recommendations in regard to the frequency or nature of spinal radiograph obtainment in AIS patients being treated in TLSO braces.

In the years following the publication of the concensus statement, the use of micro-dose radiation imaging (SterEOS 2D/3D (EOS imaging, Paris, France)), which delivers a radiation dose 5.9 to 27 times lower than a standard x-ray, has become the standard spinal imaging modality used at this institution [16, 17]. An additional benefit of this technology is the ability to generate 3D reconstructions of the spine and thoracic cage both pre-brace and in-brace when PA and lateral x-rays are obtained simultaneously. A recent study by Pasha demonstrated that 3D patient-specific parameters (lordosis, thoracic rotation, shape of the rib cage, and sagittal profile) and brace design (which allows larger in brace lordosis, better in brace Cobb correction) are important predictors of the brace effectiveness in AIS [15].

In this study, an average patient wore their TLSO brace for 16 h per day over the course of 2 years. Forty-six percent (42/90) of those patients had inconsistent in-brace radiographic documentation, and the effect of the brace on the scoliotic curve was unknown for over a year of the treatment course.

Forty-eight percent (20/42) of the inconsistently followed patients failed bracing and went on to require surgery compared to only (11/48) 23% of the patients who were followed consistently. These inconsistently followed patients were also 3.1 (95% CI 1.2-7.6) times more likely to fail bracing than patients who were consistently followed with in-brace x-rays.

Low Risser grade and an open tri-radiate cartilage are known independent risk factors for curve progression [1–6, 9, 10, 12]. This study highlights that these very skeletally immature Risser 0, open tri-radiate patients also required the longest durations of brace treatment, and were the most likely to require major brace adjustments and new brace fabrication. Unfortunately, these very immature patients were also followed less consistently with in-brace x-rays than the other patients in this study. The decreased use of radiographs in these Risser 0/open tri-radiate patients may have occurred due to a concern of radiation exposure.

**Fig. 3:**
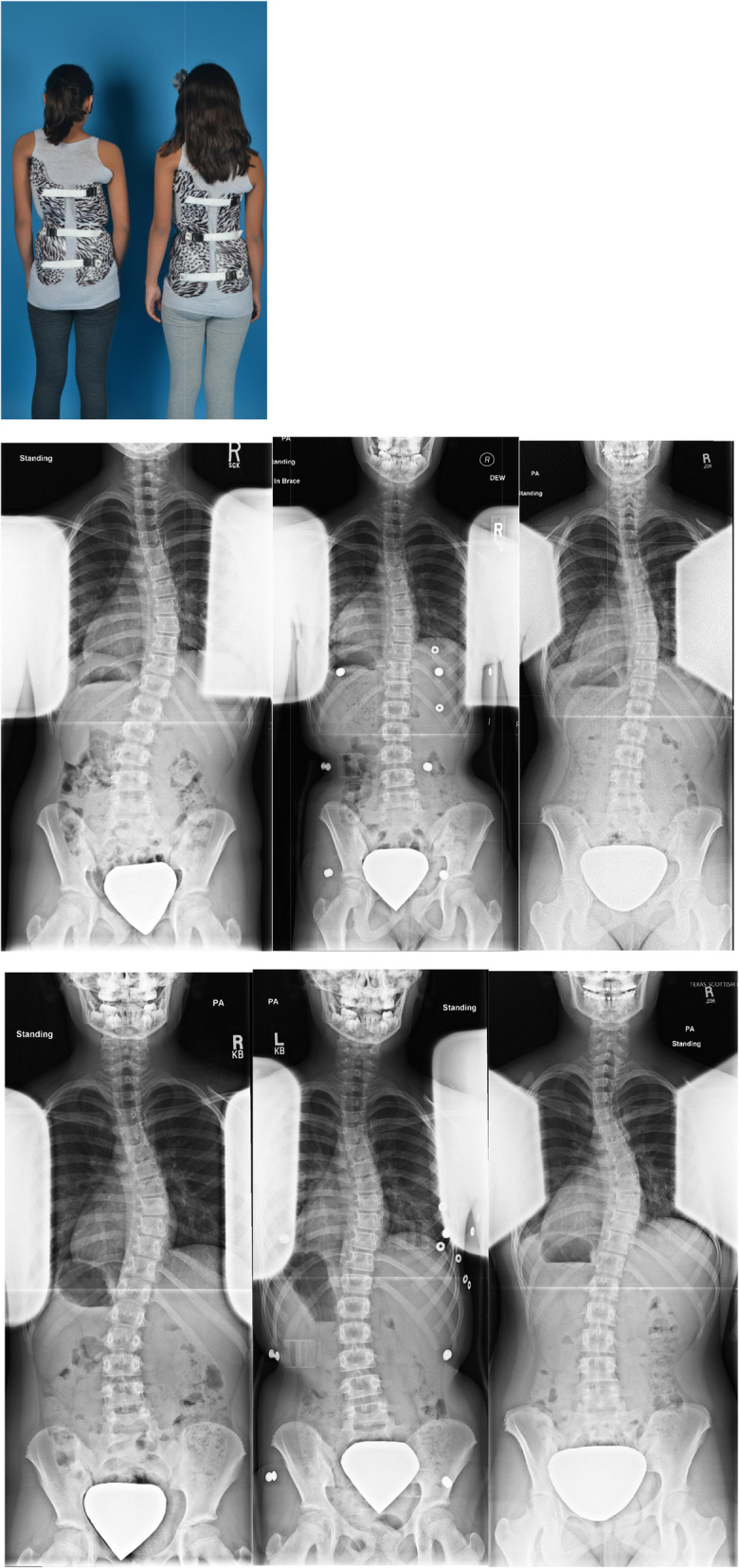
Twin sisters both Risser 0/tri-radiates open at brace initiation. Successful fulltime TLSO brace treatment

Limitations of this study include the retrospective review of the prospectively collected data and the lack of uniformity in treatment protocol among multiple physicians. Although we are using temperature monitors to verify compliance with brace wear, we were not able to follow the quality of brace wear, or how tightly the brace was worn. The largest limitation is the confounding that occurred within the very immature (Risser 0/open tri-radiate) patients also being less consistently followed with in brace x-rays while also requiring more major brace adjustments and new brace fabrications during their treatment course (Fig. 3).

The evaluation of micro-dose radiation imaging (SterEOS 2D/3D (EOS imaging, Paris, France)) allows 3D reconstructions of the spine and thoracic cage to be generated when PA and lateral x-rays are obtained simultaneously, and advancing concepts of 3D predictors of TLSO brace effectiveness should be leveraged to improve successful AIS brace treatment [18]. The AIS patients in this study wore their braces an average of 16.5 h/day for an average of 2 years (an average of 12,045 h of brace wear per patient). We advocate to ensure that the TLSO brace is effectively controlling the curve throughout the duration of treatment by obtaining new in-brace x-rays with any large brace adjustments or new brace fabrications.

## Conclusion

Obtaining new in-brace x-rays after major TLSO brace adjustments or new brace fabrication is necessary to ensure effective curve control throughout the duration of treatment. AIS patients who were inconsistently followed are 3.1 times more likely to progress to surgery. Our institution is currently implementing a standardized bracing protocol, including requiring new micro-dose in-brace PA and lateral x-rays obtained simultaneously after any large brace adjustment or new brace fabrication to ensure that the TLSO brace is effectively controlling the curve. Future prospective studies will determine the affect this has on the highest risk AIS brace patients (Risser O/tri-radiates open).

## Data Availability

The data pertaining to this study is available and accessible for review as allowed by the guidelines of our Institutional Review Board and other research regulatory entities.

## Abbreviations

TLSO: Thoraco-lumbo-sacral orthotic
AIS: Adolescent idiopathic scoliosis
BMI: Body mass index
